# Fracture prevention in COPD patients; a clinical 5-step approach

**DOI:** 10.1186/s12931-015-0192-8

**Published:** 2015-03-07

**Authors:** Elisabeth APM Romme, Piet Geusens, Willem F Lems, Erica PA Rutten, Frank WJM Smeenk, Joop PW van den Bergh, Peter ThW van Hal, Emiel FM Wouters

**Affiliations:** Department of Respiratory Medicine, Catharina hospital, PO Box 1350, 5602 ZA Eindhoven, The Netherlands; Department of Rheumatology, Maastricht University Medical Centre+, PO Box 5800, 6202 AZ Maastricht, The Netherlands; Department of Rheumatology, VU University Medical Centre, PO Box 7057, 1007 MB Amsterdam, The Netherlands; Centre of expertise for chronic organ failure (CIRO+), Research and education, PO Box 4009, 6080 AA Haelen, The Netherlands; Department of Internal Medicine, VieCuri Medical Centre, PO Box 1926, 5900 BX Venlo, The Netherlands; Department of Respiratory Medicine, Antwerp University Hospital, Wilrijkstraat 10, 2650 Edegem, Belgium; Department of Respiratory Medicine, Maastricht University Medical Centre+, PO Box 5800, 6202 AZ Maastricht, The Netherlands

**Keywords:** COPD, Osteoporosis, Fracture, Prevention, Therapy

## Abstract

Although osteoporosis and its related fractures are common in patients with COPD, patients at high risk of fracture are poorly identified, and consequently, undertreated. Since there are no fracture prevention guidelines available that focus on COPD patients, we developed a clinical approach to improve the identification and treatment of COPD patients at high risk of fracture. We organised a round-table discussion with 8 clinical experts in the field of COPD and fracture prevention in the Netherlands in December 2013. The clinical experts presented a review of the literature on COPD, osteoporosis and fracture prevention. Based on the Dutch fracture prevention guideline, they developed a 5-step clinical approach for fracture prevention in COPD. Thereby, they took into account both classical risk factors for fracture (low body mass index, older age, personal and family history of fracture, immobility, smoking, alcohol intake, use of glucocorticoids and increased fall risk) and COPD-specific risk factors for fracture (severe airflow obstruction, pulmonary exacerbations and oxygen therapy). Severe COPD (defined as postbronchodilator FEV_1_ < 50% predicted) was added as COPD-specific risk factor to the list of classical risk factors for fracture. The 5-step clinical approach starts with case finding using clinical risk factors, followed by risk evaluation (dual energy X-ray absorptiometry and imaging of the spine), differential diagnosis, treatment and follow-up. This systematic clinical approach, which is evidence-based and easy-to-use in daily practice by pulmonologists, should contribute to optimise fracture prevention in COPD patients at high risk of fracture.

## Introduction

COPD is a major cause of morbidity and mortality worldwide and results in a high and steadily increasing economic and social burden. The prevalence of COPD is approximately 9 to 10% in adults aged 40 or older [1]. COPD was the fifth leading cause of death in the world in 2002, and is projected to be the third leading cause of death by 2030 [2].

COPD is being considered as a complex and heterogeneous disease with clinically significant comorbidities [3,4]. These comorbidities are more often seen in both COPD patients and smokers than in non-smokers [3,5]. The majority of COPD patients has 4 or more comorbidities [6], of which hypertension, cardiovascular diseases, gastro-oesophageal reflux, depression, anxiety and osteoporosis are most frequently found [7].

A meta-analysis stated that the overall prevalence of osteoporosis in COPD is 35% [8]. A cross-sectional study with 27 patients with moderate COPD, 45 patients with severe COPD and 13 patients with very severe COPD (92% men, mean age 75 years) demonstrated that 35% of patients had 1 or more radiographic vertebral fractures [9]. In this study, patients with postbronchodilator FEV_1_ < 30% predicted were at higher risk of vertebral fracture compared with patients with FEV_1_ ≥ 50% predicted (p = 0.02). A cross-sectional study with 3030 COPD patients (58% men, mean age 70 years) showed that 35% of patients with mild COPD, 38.5% of patients with moderate COPD, 45.7% of patients with severe COPD and 59.2% of patients with very severe COPD had 1 or more vertebral fractures [10]. In 87360 men with COPD (mean age 67 years, no data on COPD severity reported), the hip fracture rate was 4.0 per 1000 person years and the wrist fracture rate was 1.3 per 1000 person years during a follow-up of 2.7 years [11]. Furthermore, a cross-sectional study with 71 patients with mild COPD, 100 patients with moderate COPD, 57 patients with severe COPD and 27 patients with very severe COPD (62% men, mean age 68 years) showed that 51% of patients had osteoporosis, defined as a T-score ≤ −2.5 or a prevalent vertebral fracture [12]. However, the majority (80%) of patients with osteoporosis was not treated with anti-osteoporosis medication.

Osteoporosis-related fractures may contribute to increased morbidity and mortality in COPD. Indeed, vertebral fractures may further reduce the already compromised pulmonary function in COPD [13] and hip fractures may increase mortality due to a higher operation risk related with COPD [14]. Since COPD patients with increased fracture risk are poorly identified, and consequently, undertreated [12], we propose a 5-step clinical approach [15] to improve identification of COPD patients at high risk of fracture by taking into account both classical and COPD-specific risk factors for fracture.

## Review

### Working group composition

A round-table discussion was organised with 8 clinical experts in the field of COPD and fracture prevention (3 pneumologists, 2 rheumatologists, 1 endocrinologist and 2 clinical researchers) in the Netherlands in December 2013. Three of them were involved in the last revision of the Dutch guideline on fracture prevention in 2011. The Dutch guideline was developed by, and developed for, all groups involved in fracture prevention (general practitioners, specialists, nurses and patients’ organisations). Every member of the working group studied the literature over the past 10 years on the following topics: the prevalence of osteoporosis and fractures in COPD, classical and COPD-specific risk factors for osteoporosis and fracture, the role of oral and inhaled glucocorticoids on osteoporosis and fracture, and fracture prevention. The literature research was discussed during the round-table discussion with particular focus on the following topics: COPD-specific risk factors for osteoporosis and fracture, and introduction of a systematic clinical approach for fracture prevention in COPD.

### Risk factors for fracture in COPD

Classical risk factors for osteoporosis and fracture include low body mass index (BMI), older age, previous fracture before the age of 50, parent with a fracture, immobility, current smoking, alcohol intake (3 or more units a day), use of glucocorticoids, secondary osteoporosis and increased fall risk [16]. In addition to these classical risk factors, there are several COPD-specific risk factors for osteoporosis and fracture, including severe airflow obstruction [17,18], emphysema, exacerbations and oxygen therapy [8-11,19]. In accordance to the flowchart of Lehouk and colleagues [20], severe COPD (defined as postbronchodilator FEV_1_ < 50% predicted) was included as a supplementary risk factor in our clinical approach for fracture prevention. Other COPD-specific risk factors were not included because of lack of thresholds for inclusion as a risk factor.

#### Oral glucocorticoids

Oral glucocorticoids are dose-dependently related with a decrease in bone mineral density (BMD) and increase in fracture risk [21,22]. In patients who use oral glucocorticoids, the relative risk of a vertebral fracture is 2.6 (2.3–2.9), a non-vertebral fracture 1.3 (1.3-1.4), a hip fracture 1.6 (1.5-1.8) and a wrist fracture 1.1 (1.0-1.2) [22]. Fracture risk increases immediately after starting glucocorticoid therapy and decreases quickly after stopping glucocorticoid therapy [22].

#### Inhaled glucocorticoids

Inhaled glucocorticoids were developed to reduce systemic side-effects. Since part (10 to 40%) of the inhaled glucocorticoids that reaches the lungs will also come into the systemic circulation via the bronchial circulation, inhaled glucocorticoids may still cause some systemic side-effects [23,24]. Pouw and colleagues [25] demonstrated that the use of inhaled beclometason was related with decreased serum osteocalcin concentrations that may contribute to reduced bone formation.

Data on the systemic clinical effects of inhaled glucocorticoids have been conflicting. A Cochrane review of the literature showed that inhaled glucocorticoids had no effect on BMD or fracture risk during a follow-up of 3 years [26]. However, a meta-analysis of 16 randomised controlled trials and 7 observational studies showed that inhaled glucocorticoids were related with a significant increased risk of fracture (OR 1.27 (1.01 – 1.58)) during a mean trial duration of 90 weeks [27]. An increase of 500 mcg beclometason equivalents per day was related with a 9% increased risk of fracture (1.1 (1.06-1.12)) [27].

#### Systemic inflammation

Systemic inflammation might be one of the key components in the development of COPD-related comorbidities [28]. Inflammatory markers, such as C-reactive protein, fibrinogen, tumor necrosis factor-alpha, interleukin-6 and interleukin-8, have been shown to be increased in the peripheral blood in COPD patients, compared with smokers who have not developed the disease [29,30]. Systemic inflammation might be the result of a systemic “spill-over” of inflammatory mediators from local lung inflammation [31], or might represent a systemic component of the disease that develops in parallel with, or prior to, pulmonary inflammation [32]. Tobacco smoking, tissue hypoxia, infections and exacerbations might also contribute to systemic inflammation.

Increased systemic inflammation is related with the presence of several COPD-related comorbidities, including osteoporosis [32]. Systemic inflammation might cause abnormalities in the balance and rate of bone remodeling via disturbances in the RANK/RANKL/OPG pathway [33-35]. These abnormalities in bone remodelling might consequently result in bone fragility and increased fracture risk. Clinical studies confirming a causal relationship between systemic inflammation and bone alterations in COPD patients have not been published yet.

Since glucocorticoids are prescribed to decrease lung inflammation, they may be expected to inhibit systemic inflammation and hence exhibit some positive effects on bone. In patients with rheumatoid arthritis, therapy with methotrexate and prednisone 10 mg per day led to decreased disease activity but did not reduce BMD compared with methotrexate monotherapy [36]. In 176 patients with bronchitis, therapy with low dose inhaled glucocorticoids was related with a reduced loss of BMD compared with therapy without inhaled glucocorticoids (0.002 g/cm^2^/year versus 0.006 g/cm^2^/year, p = 0.02) [37].

#### Repair processes

A pathway that has been related with both COPD and osteoporosis is the Wnt/β-catenin signaling pathway. On the one hand, it plays a role in bone remodeling and repair of micro fractures, while on the other hand, it is involved in lung epithelial injury and repair processes [38]. Therefore, impairment of Wnt/β-catenin signaling might result in both COPD and osteoporosis.

### A systematic approach for fracture prevention in COPD

**Figure 1:**
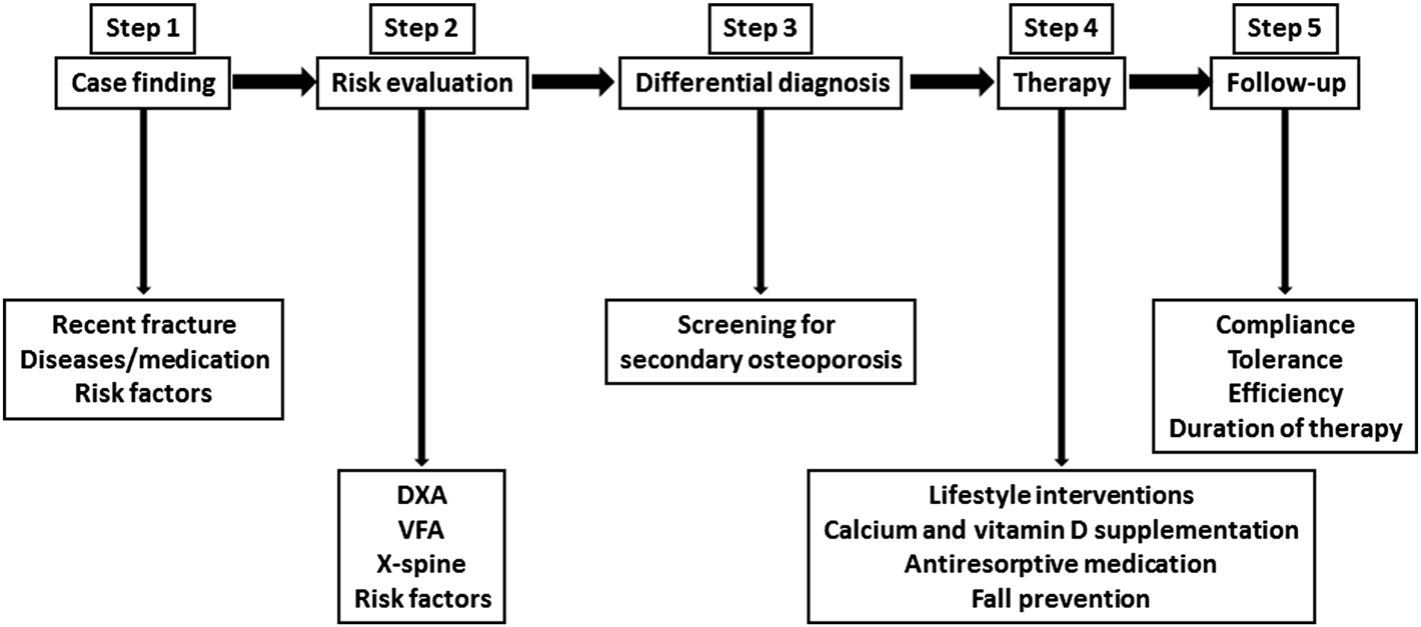
**A multistep evaluation and treatment plan for fracture prevention in COPD.** DXA = dual energy X-ray absorptiometry of hip and lumbar spine; VFA = vertebral fracture assessment with DXA**.**

**Figure 2:**
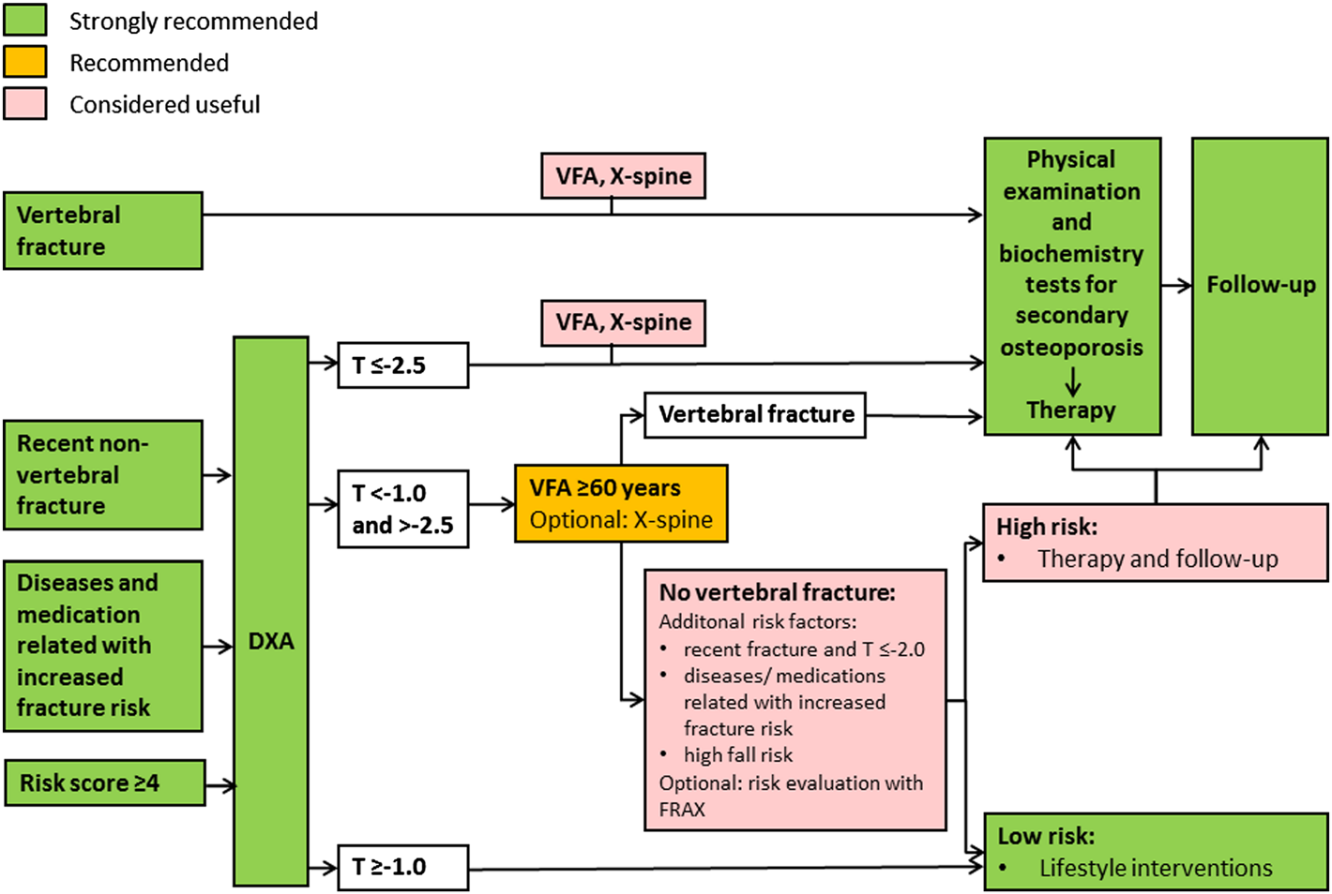
**Evaluation of fracture risk in COPD patients.** DXA = dual energy X-ray absorptiometry of hip and lumbar spine, FRAX = fracture risk assessment tool, VFA = vertebral fracture assessment with DXA, X-spine = X-ray of the spine**.** This figure is based on the Dutch guideline on osteoporosis and fracture prevention [16].

**Figure 3:**
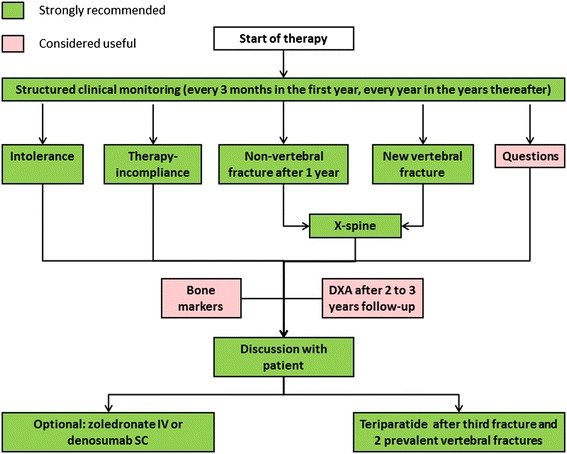
**Flowchart for follow-up.** This figure is based on the Dutch guideline on osteoporosis and fracture prevention [16].

**Figure 4:**
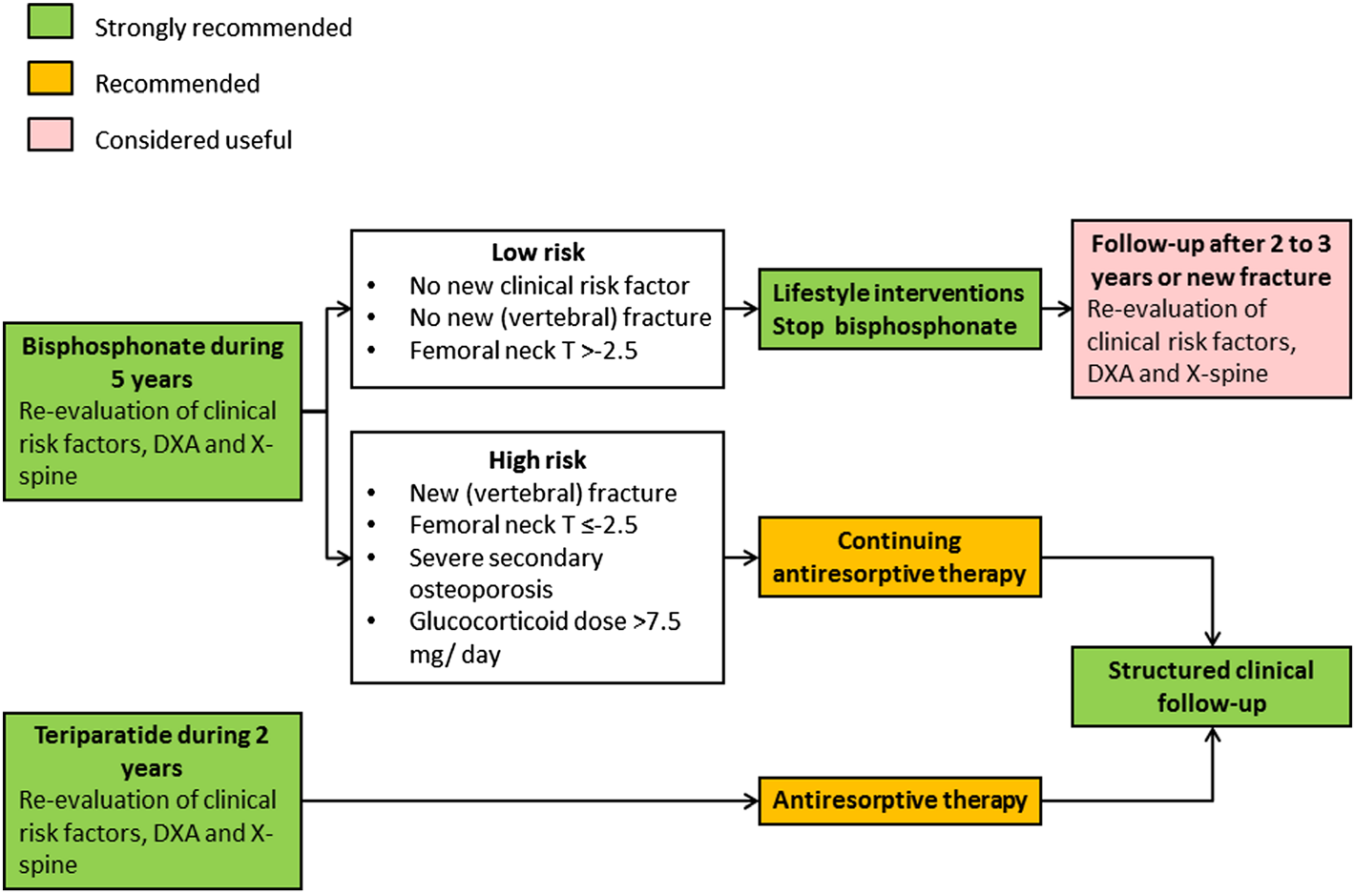
**Re-evaluation after 5 years of treatment with bisphosphonate or 2 years of treatment with teriparatide.** This figure is based on the Dutch guideline on osteoporosis and fracture prevention [16].

Since COPD patients with increased fracture risk are poorly identified and undertreated, we propose a systematic evaluation and treatment plan for fracture prevention in COPD. This 5-step clinical approach starts with clinical case finding, followed by risk evaluation, differential diagnosis, treatment and follow-up (Figure 1). In each step, recommendations are graded as “strongly recommended”, “recommended” and “considered useful” based on the strength of evidence (Figures 1,2,3 and 4) [15,16,20].

#### Step 1: Case finding

In case doctors or patients have questions about fracture risk, COPD patients who are 50 years or older are recommended to be assessed for fracture risk using the COPD-adapted risk score (Table 1). This COPD-adapted risk score is based on the revised Dutch guideline on fracture prevention [16] and the flowchart of Lehouck and colleagues [20].

**Table 1:** **COPD-adapted risk score**

**Risk factor**	
Weight <60 kg or BMI <20 kg/m^2^	1
Age >60 years	1
Age >70 years (in this case do not count risk score ‘age >60 years’)	2
Previous fracture after the age of 50 (more than 2 years ago)	1
Parent with hip fracture	1
Immobility	1
Rheumatoid arthritis	1
More than 1 fall in the last year	1
Diseases or medications associated with increased fracture risk*	1
Alcohol ≥3 units per day	1
COPD and postbronchodilator FEV_1_ < 50% predicted	1

*Diseases or medications associated with increased fracture risk: untreated hypogonadism, inflammatory bowel diseases or malabsorption, chronic inflammatory diseases, organ transplantation, diabetes mellitus, untreated hyperthyroidism or over substituted hypothyroidism, primary hyperparathyroidism, pernicious anaemia and any past or current use of oral glucocorticoids for at least 3 months.

COPD-adapted risk score ≥4 represents increased fracture risk.

COPD patients who are 50 years or older and fulfil one or more of the criteria below are advised further fracture risk evaluation as described in step 2:

1. A prevalent non-traumatic vertebral fracture,
2. A recent non-vertebral fracture (during the last 2 years),
3. Current use of glucocorticoids (≥7.5 mg prednisone equivalents per day during at least 3 months),
4. A COPD-adapted fracture risk score ≥4 (Table 1).

#### Step 2: Risk evaluation

The above mentioned COPD patients are advised further fracture risk evaluation, which includes dual energy X-ray absorptiometry (DXA) of the hip and spine, imaging of the spine and evaluation of clinical risk factors (Figure 2).

The gold standard for diagnosing osteoporosis is DXA. For diagnostic purposes, osteoporosis is defined as a BMD 2.5 standard deviations or more below the reference mean (T-score ≤ −2.5) and osteopenia as a BMD 2.5 to 1.0 standard deviations below the reference mean (T-score between −1.0 and −2.5). Patients with osteoporosis of the hip or spine and patients with osteopenia who have additional risk factors, such as a newly diagnosed vertebral fracture or important clinical risk factors, are candidates for fracture prevention.

Since vertebral fractures are related with increased fracture risk independently of BMD [39], imaging of the thoracic and lumbar spine is advocated for fracture risk evaluation and can be performed by conventional radiography or DXA-based lateral images of the spine (vertebral fracture assessment [VFA]). If lateral X-rays of the thorax are available for evaluating lung involvement, pulmonologists are in a privileged position to evaluate these images for the presence of vertebral fractures, as they are often overlooked or not reported by radiologists [40]. Vertebral fractures are classified according to Genant’s visual semi-quantitative method into wedge, biconcave or crush and into grade 1 (20 to 25% reduction in height and 10 to 20% reduction of the projected vertebral area), grade 2 (25 to 40% reduction in height and 20 to 40% reduction of the projected vertebral area) and grade 3 (more than 40% reduction in height and area) [41].

The fracture risk assessment tool (FRAX) (https://www.shef.ac.uk/FRAX/tool.aspx) might be additionally helpful to select patients who benefit from fracture prevention therapy. However, the FRAX tool has some disadvantages. COPD, physical inactivity and increased risk of falls have been related with osteoporosis and fractures, but are not included in the FRAX model. In addition, longitudinal studies with COPD patients using the FRAX tool for fracture prediction are not yet available.

Based on fracture risk evaluation, the following patients are at high risk of fracture and are advocated to consider fracture prevention:

1. A prevalent non-traumatic vertebral fracture,
2. Osteoporosis in the spine or hip,
3. Osteopenia with additional risk factors, such as a newly diagnosed vertebral fracture or important clinical risk factors (e.g. increased fall risk or high FRAX score).

#### Step 3: Differential diagnosis

In COPD patients, the following causes of secondary osteoporosis and other metabolic bone diseases are frequently documented: vitamin D deficiency (58%) [42], secondary hyperparathyroidism (10%) [43], renal insufficiency (22%) [6] and hypogonadism (22-69%) [44]. Therefore, screening for the presence of secondary osteoporosis or other metabolic bone diseases is advocated. Screening should exist of medical history, physical examination and biochemistry tests, including serum calcium, phosphate, creatinine, albumin, erythrocyte sedimentation rate, 25-hydroxyvitamin D, 24 h urine calcium in men, and serum testosterone in men younger than 70 years [45]. Additional tests may be needed based on clinical and biochemistry findings in order to detect hyperparathyroidism, multiple myeloma, hypercortisolism, celiac disease, or other causes of secondary osteoporosis and metabolic bone diseases. These results should be taken into account and treated when possible before starting fracture prevention.

#### Step 4: Therapy

Fracture prevention starts with advising a healthy lifestyle, including quit smoking, physical exercise (walking during 30 minutes 3 times a week), limiting alcohol intake and healthy diet with both sufficient calcium (total intake of 1000 to 1200 mg per day) and vitamin D [46,47]. If dietary calcium intake is insufficient (less than 1000 mg per day), calcium supplementation is recommended. As an example: a patient who does not use any dairy products needs 1000 mg calcium supplementation per day; a patient who uses 1 to 2 dairy products per day needs 500 mg calcium supplementation per day; and a patient who uses 3 or more dairy products per day does not need any calcium supplementation. Calcium supplementation does not need to be higher, as a too high calcium intake could be related with an increased risk of cardiovascular events [48,49]. To optimise calcium homeostasis, sufficient vitamin D is necessary. A dose of 800 IU vitamin D per day is advocated [16,50]. In COPD patients with a history of >1 falls in the last year, fall prevention is advocated [51]. Exacerbations of COPD might contribute to bone loss via increased systemic inflammation, use of corticosteroids and reduced physical activity. Therefore, (chest) physicians should consider carefully the prescription of oral glucocorticoids in each individual patient and they should support patients to keep moving. More research is however necessary to investigate the impact of these isolated components related with exacerbations on bone metabolism.

Drug treatment includes two categories of drugs: antiresorptive medications such as bisphosphonates or denosumab and osteo-anabolics such as the 1–34 rhPTH fragment teriparatide. In the Dutch guideline [16], alendronate and risedronate are recommended as first choice medications because of their broad-spectrum fracture prevention and generic availability. Denosumab and zoledronate are indicated in case of intolerance for oral bisphosphonates. Teriparatide is indicated in severe osteoporosis, i.e. patients who develop new fractures in spite of adequate treatment with antiresorptive drugs. Notably, the clinical effects of these medications have not been studied in randomised controlled trials with COPD patients.

#### Prevention of glucocorticoid induced osteoporosis

Prevention of glucocorticoid induced osteoporosis depends on the background risk and the dose and duration of glucocorticoid treatment [16]. Bisphosphonates are advocated in: 1, patients who use more than 15 mg prednisone equivalents per day; 2, patients who had a fracture after the age of 50 years and use, or are going to use, oral glucocorticoids for 3 or more months; and 3, postmenopausal women and men older than 70 years who use 7.5 to 15 mg prednisone equivalents per day. In premenopausal women and men younger than 70 years who use 7.5 to 15 mg prednisone equivalents per day and in patients who use less than 7.5 mg prednisone equivalents per day, a low BMD is an indication for treatment, although no consensus exists about the level of T-score for treatment decision (T-score < −1.0 or < −1.5).

#### Step 5: Follow-up

The aim of follow-up is to check adherence and tolerance and to decide about efficacy and duration of therapy. As adherence to fracture prevention medication is low, regular follow-up is advocated, e.g. 3 months after start of treatment, and then yearly. In case of intolerance for oral bisphosphonates, treatment can be switched to IV zoledronate or SC denosumab. In case of a new fracture after one year of adequate antiresorptive drug therapy, teriparatide is advocated during 2 years (Figure 3).

After 5 years of treatment with bisphosphonates, re-evaluation of clinical risk factors, BMD and imaging of the spine should take place (Figure 4). If fracture risk is low, treatment can be interrupted, with re-evaluation after 2 to 3 years. If fracture risk is still high, antiresorptive treatment can be continued, with re-evaluation after 5 years. After treatment of teriparatide, antiresorptive drugs are advocated to conserve the gain in bone density and structure.

### Limitations and future research directions

In COPD patients, there are no guidelines available on the necessity or frequency of serial DXA scanning and vertebral fracture assessments. Graat-Verboom and collegues [52] studied the incidence of osteoporosis during 3 years follow-up. The prevalence of osteoporosis increased from 47% to 61% in 3 years. The increase in osteoporosis was mostly due to newly diagnosed vertebral fractures. In addition, osteopenia at the hip seemed to be a predictor of osteoporosis. Based on their data, Graat-Verboom and colleagues [52] advised to perform vertebral fracture assessments in all COPD patients every year and to perform DXA scanning in patients with osteopenia at the hip every 3 years. Since this is the only longitudinal evaluation of osteoporosis in COPD patients, additional research is necessary to provide guidelines on the necessity and frequency of serial DXA scanning and vertebral fracture assessments.

Although the 5-step clinical approach for fracture prevention has been based on the current literature on osteoporosis in COPD, it has not been validated yet. Therefore, future research should be focussed on algorithms to identify COPD patients at high risk of fracture and beneficial effects of anti-fracture therapy in COPD patients.

## Conclusions

Although osteoporosis and its related fractures are common in patients with COPD, patients with increased fracture risk are poorly identified and undertreated. Here, we described a clinical systematic approach for fracture prevention in COPD patients starting with clinical case finding, followed by risk evaluation, differential diagnosis, treatment and follow-up.

## Abbreviations

BMD: Bone mineral density
BMI: Body mass index
COPD: Chronic obstructive pulmonary disease
DXA: Dual energy X-ray absorptiometry
FRAX: Fracture risk assessment tool
VFA: Vertebral fracture assessment with dual energy X-ray absorptiometry
X-spine: X-ray of the spine
